# Prevalence, Predictors and Mechanisms of Steam Pops in Ablation Index-Guided High-Power Pulmonary Vein Isolation

**DOI:** 10.3390/jcdd9120441

**Published:** 2022-12-07

**Authors:** Tian Shuang, Lingcong Kong, Fuyu Cheng, Xinhua Wang

**Affiliations:** Department of Cardiology, Renji Hospital, Shanghai Jiao Tong University School of Medicine, Shanghai 200080, China

**Keywords:** steam pop, high-power circumferential pulmonary vein isolation, radiofrequency, tissue coverage, delta impedance

## Abstract

Despite the good cooling effect of the contact-force porous catheter, the risk of steam pops (SP) remains one of the major concerns in high-power circumferential pulmonary vein isolation (CPVI). This study aimed to investigate the prevalence, predictors and possible mechanisms of SPs in CPVI. Patients experiencing SPs in de novo high-power CPVI were 1:3 matched by non-SP patients with gender, age (±5 years) and left atrial diameter (LAD) (±5 mm) to compare the ablation parameters of SP and non-SP lesions. Catheter tip displacement (Tip_disp_) was compared between “edge-of-ridge” and “PV-side-of-ridge” placement at anterior and roof segments of the left pulmonary vein (PV). SPs occurred in 11 (1.57%) of 701 patients, including 6 at the antero-superior left PV, 2 at the roof, 1 at the postero-superior left PV, 1 at the bottom left PV and 1 at the antero-superior aspect of the right PV. There was significantly shorter RF delivery duration (13.9 ± 6.3 vs. 23.3 ± 6.0 s), greater Δimpedance (17.6 ± 6.7 vs. 6.7 ± 4.1 Ω) and lower ablation index (357.7 ± 68.8 vs. 430.2 ± 30.7) in SP patients than those in non-SP patients. Δimpedance >12 Ω during ablation could predict SP occurrence. Tip_disp_ was greater in “PV-side-of-ridge” than that in “edge-of -ridge” placement (3.2 ± 1.6 mm vs. 2.0 ± 0.8 mm) at antero-superior and roof segments of the left PV. The prevalence of SP was 1.57% in high-power CPVI procedures, with the most common site at the antero-superior segment of the left PV. Δimpedance was a significant predictor of SP occurrence. “PV-side-of-ridge” ablation at antero-superior and roof segments of left PV might predispose to SP occurrence due to excessive tissue coverage.

## 1. Introduction

Circumferential pulmonary vein isolation (CPVI) is an established approach for paroxysmal atrial fibrillation (PAF) ablation, and the mainstay approach for persistent AF (PeAF) ablation [1]. In the past decade, the contact-force sensing, saline-irrigated radiofrequency (RF) catheter was introduced in clinical practice with better efficacy and safety than the conventional catheters [2,3,4]. Recently, the porous tip of the catheter was upgraded from a 6-hole to 56-hole design. The latter provides a more powerful cooling of the surrounding tissue, whereby a high power (50–70 W) is applied to shorten the CPVI procedure [5,6,7,8].

Despite the good tissue-cooling effect of the contact-force 56-hole catheter, the risk of steam pops (SP) remains one of the major concerns in CPVI procedures, especially when high RF power is delivered [6,7]. SPs result from steam explosion when the tissue temperature exceeds the boiling point during RF ablation. They might be uneventful, but could lead to severe pericardial effusion or tamponade which necessitates pericardiocentesis/drainage, or even surgical repair [9]. However, the prevalence and predictors of SPs have not been fully clarified, and the cause of SP remains undetermined.

This study sought to investigate the prevalence and predictors of SPs in high-power CPVI and explore the underlying mechanisms of SPs in a consecutive cohort of patients with PAF or PeAF.

## 2. Methods

### 2.1. Patients’ Population

**Figure 1:**
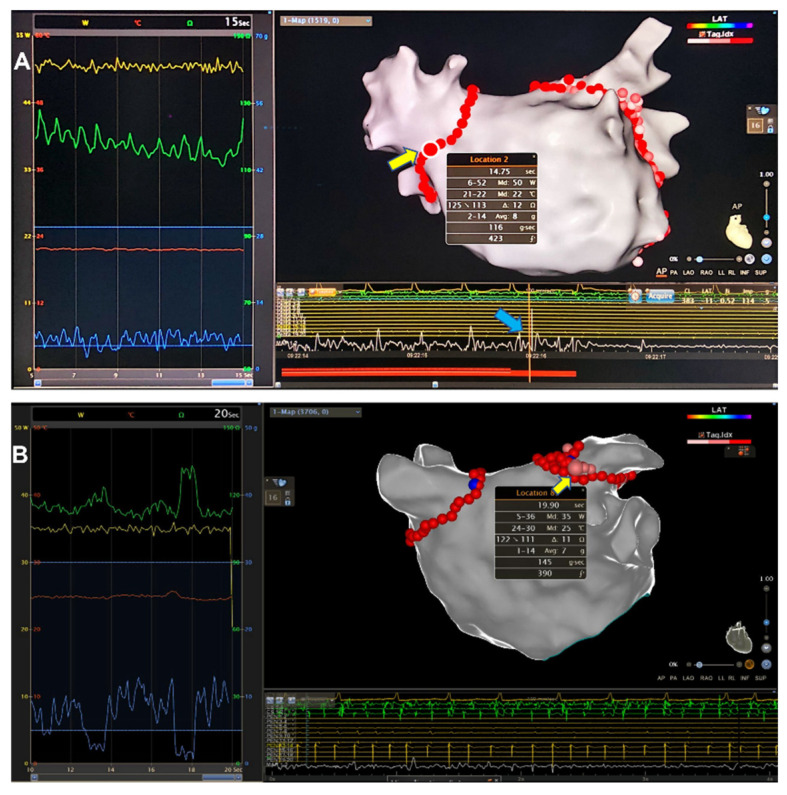
**Two examples of steam pops (SP) occurring in CPVI**. (**A**) An audible SP occurred at the right antero-superior aspect (yellow arrow) of right-sided PV’s antrum when RF energy was delivered for 15 s (50 W, median CF 8 g (maximum 14 g), AI 423). Note the impedance decreased from 125 to 113 Ω and surged immediately before SP occurrence, along with a high-amplitude and high-frequency noise signal (blue arrow) recorded on the bipolar endocardial electrograms. (**B**) An audible SP occurred at the antero-superior segment of LSPV when RF ablation lasted for 20 s (35 W, median CF 7 g (maximum 14 g), AI 390). The impedance value was fluctuating until a slope (delta impedance 11 Ω) was noted immediately before SP occurrence. However, the interference noise was insignificant on endocardial recordings. CPVI, circumferential pulmonary vein isolation; CF, contact force; RF, radiofrequency; AI, ablation index; LSPV, left superior pulmonary vein.

The patients experiencing audible SPs in de novo CPVI were enrolled from the pool of AF patients in Renji Hospital, Shanghai Jiao Tong University School of Medicine between August 2020 and March 2022. An audible SP was defined as an explosive sound frequently concomitant with high-frequency noise signal on the endocardial recordings and abrupt impedance change (Figure 1). Each SP patient was 1:3 matched by non-SP patients with gender, age ±5 years old and left atrial diameter (LAD) ±5 mm (measured by transthoracic echocardiography [TTE]). Transesophageal echocardiography (TEE) was applied to exclude left atrial thrombi. Patients with prior catheter or surgical ablation, history of vein Marshall ethanol infusion and absence of PV isolation during AF ablation were excluded. To explore the underlying mechanisms of SPs, another cohort of non-SP AF patients (*n* = 11) having matched gender, age (±5 years old) and LAD (±5 mm) with the SP patients was included to compare the extent of catheter tip displacement from a contact force (CF) of 5 g to 10 g between the “edge of ridge” and the “PV-side of ridge” placement. All patients provided written informed consent. The study was approved by the Institutional Ethic Committee of Renji Hospital.

### 2.2. Electrophysiological Study

The procedure was performed under conscious sedation and analgesia with continuous infusion of fentanyl and midazolam. A decapolar mapping catheter was positioned in the coronary sinus (CS) (Abbott Medical, Abbott Park, IL, USA) via left femoral vein access. Two SL1-type Swartz sheathes (Fast-Cath^TM^, Abbott Medical, Abbott Park, IL, USA) were inserted from the right femoral vein and introduced in the left atrium (LA) by two transseptal punctures. Heparin 100 U/kg were infused through the sheath and superadded 1000 U every 1 h to maintain an activated clotting time of 300–350 s. A duo-decapolar mapping catheter (PentaRay, Biosense Webster, Irvine, CA, USA) was advanced through the sheath for creation of LA geometry and recording of pulmonary vein (PV) potential. A 56-hole contact-force porous ablation catheter (Thermocool SmartTouch^®^ SF, Biosense Webster, Irvine, CA, USA) was used for CPVI.

### 2.3. Segmentation of Circular Lesion Line and Comparison of SP Parameters

**Figure 2:**
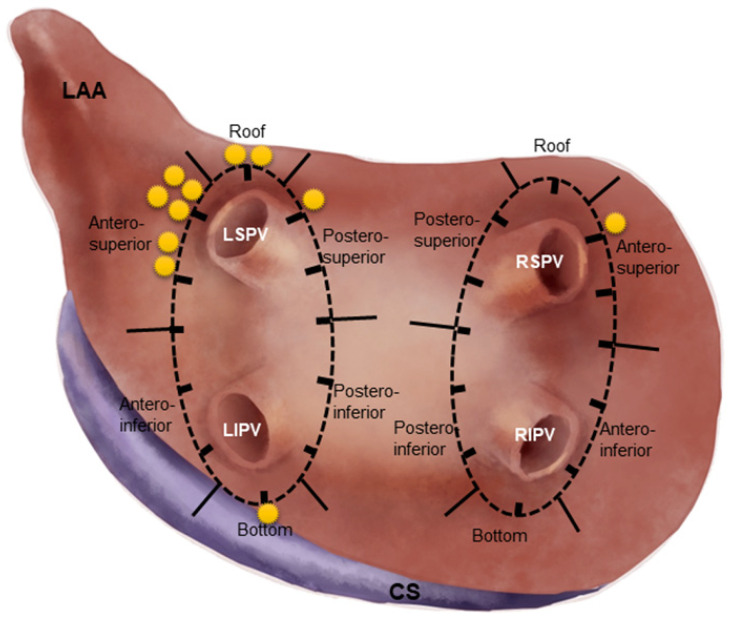
**Anatomic distribution of SPs in circular PV lesion lines.** The circular lesion line was divided into 6 segments for bilateral PVs, and each asterisk represented one SP. Note: the most common site of SPs was at the antero-superior segment of LPV lesion line. LPV, left pulmonary vein.

To describe the sites of SPs, the lesion line encircling ipsilateral PVs was firstly divided into 6 segments: roof, bottom, antero-superior (AS), antero-inferior, postero-superior (PS) and postero-inferior (PI). Then the circular lesion line was viewed as the clock dial, on which a 1 to 12 o’clock direction was designated (Figure 2). When an SP occurred, the location and the ablation parameters (RF power, duration, CF, ablation index (AI) and impedance change (Δimpedance) were documented. The ablation parameters at SP sites were compared with those at the 1:3 matched sites in non-SP patients.

### 2.4. High-Power CPVI and Management in Case of SP Occurrence

The CPVI procedure was well-established and described in detail elsewhere [10]. Briefly, the four PVs, PVs’ ostia and the LA geometry were reconstructed by roving the PentaRay in each PV and at every aspect of the LA. Lesions were created ≈1–2 cm away from the PVs’ ostia to reduce the risk of PV stenosis. To stabilize the catheter and counteract respiratory and cardiac motion, lesions were placed at the PV-side of the lateral ridge at the AS aspect of left superior PV. RF energy was delivered at 40–50 W/AI 430–450 for creating anterior/roof lesions, and at 40 W/AI 380–400 for posterior/bottom lesions in the power-controlled mode (without ramp), with the saline flow rate of 17 mL/min. The tool of automatic lesion tagging (VisiTag^®^, Biosense Webster, Irvine, CA, USA) was applied with the following settings: lesion size of 4 mm, 2.5 mm stability for 3 s and minimal contact force (CF) of 5 g for >30% time. The automatic cut-off of impedance was set for <40 Ω/0.5 s. The endpoint of CPVI was disappearance of PV potentials in all PVs or dissociation of PV potentials with atrial electrograms at the end of 20 min observation duration.

When an SP occurred, RF ablation was suspended immediately, and the patient’s vital signs, fluoroscopic cardiac motion, TTE-detected pericardial effusion, as well as symptoms of the nerve system were closely monitored for 30–60 min. If no event occurred, then RF ablation was restarted to complete the circular line until PV isolation was achieved. In the case of cardiac tamponade, RF ablation was withdrawn and emergent pericardiocentesis was performed.

### 2.5. Comparison of Tip Displacement between the “Edge of Ridge” and the “PV-Side of Ridge” Placement

**Figure 3:**
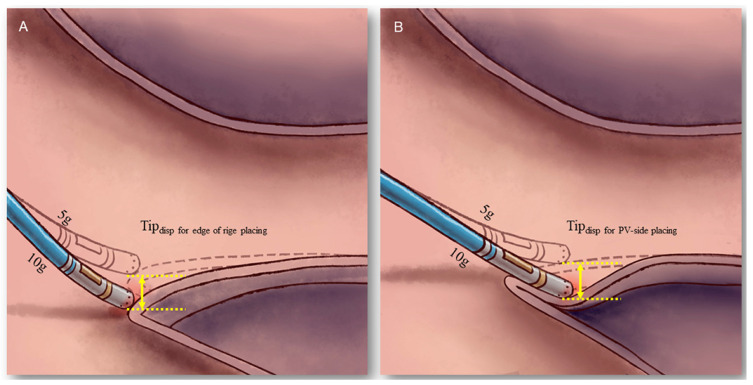
**Schematic plot illustrating different Tip_disp_ by “edge-of-ridge” and “PV-side-of-ridge” placement**. The Tip_disp_ by “edge-of-ridge” placement (**A**) was less than that by “PV-side-of-ridge” placement (**B**). This might be due to the tissue compliance at the “rigid” PV-ridge junction being inferior to that in the PV. The catheter tip interposed in the venous wall by “PV-side-of-ridge” placement (**B**), indicating more prominent tissue coverage than by “edge-of-ridge” placement.

The extent of the catheter tip displacement was measured in two means of catheter tip placement (Figure 3). The “edge of ridge” placement meant the catheter tip was positioned at the edge of the lateral ridge, while the “PV-side of ridge” placement meant it was positioned at the proximal left superior PV adjacent to the ridge. For both means of catheter placement, the location of catheter tip (Tip_loc_) was determined at the end-expiratory phase at the CF of 5 g and 10 g, respectively. The catheter tip displacement (Tip_disp_) was defined as the distance between Tip_loc_ at CF 10 g and Tip_loc_ at CF 5 g, and was measured by the built-in software toolkit of CARTO 3 system (Biosense Webster, Irvine, CA, USA). For each patient, the Tip_disp_ value was calculated at 9:00, 10:30 and 12 o’clock direction of the left PV circular line, and was compared between the “edge of ridge” and the “PV-side of ridge” placement.

Tip_disp_ was viewed as a surrogate parameter for the evaluation of tissue coverage during CPVI, because a higher Tip_disp_ indicated more catheter tip contact with the tissue and more tissue coverage over the catheter tip (Figure 3). As illustrated in Figure 3, a small artificial “pouch” was created if the catheter tip excessively interposed in the tissue, which was subject to SP occurrence during ablation [11].

### 2.6. Post-Procedural Management and Follow-Up

TTE was performed 3–4 h post-ablation and repeated 24 h later to rule out delayed pericardial effusion or tamponade. CT scan was performed after 48 h post-ablation to exclude cerebral embolism when suspected. All the patients were discharged 3 days after the procedure and followed up at the outpatient clinic regularly. If not contraindicated, oral anticoagulation with NOACs was administered for at least 3 months, and continued in patients with high thromboembolic risks. Antiarrhythmic drug therapy was administered for 2 months post-ablation, and was discontinued in those free of AF recurrence.

### 2.7. Statistical Analysis

Continuous variables with normal distribution were given as mean ± deviation, and compared by Student’s *t*-test if the variance were equal; or as median (1st quartile, 3rd quartile), and compared by Mann–Whitney U test otherwise. Category variables were described as counts or proportions, and compared by Chi-square test or Fisher’s exact test. The factors with a *p*-value < 0.1 in univariate analysis were included in multivariate analysis. Multivariate binary logistic regression analysis was performed to evaluate the procedural predictors for SPs (described as odds ratio (OR) and 95% confidence interval (CI). Area under the curve (AUC), sensitivity, specificity and cut-off value of the predictors were calculated by receiver operating characteristic (ROC) curve analysis. A two-tailed *p* value < 0.05 was considered statistically significant. Data analysis was performed by SPSS 19.0 software (IBM Corporation, Somers, NY, USA).

## 3. Results

### 3.1. Prevalence of SP in High Power CPVI

From August 2020 to March 2022, a total of 701 patients (413 males, average age 66.6 ± 9.1 years old) with PAF or PeAF were enrolled to undergo de novo catheter ablation. SPs occurred in 11 (1.57%) patients (all with a single SP). Thirty-three patients with matched gender, age and LAD were selected as the control group. There was no significant difference in baseline characteristics, except that the proportion of heart failure was higher in SP patients than in non-SP patients. Another 11 matched patients were enrolled to evaluate catheter tip displacement ahead of PV ablation. The baseline demographic data were compared in Table 1.

### 3.2. Distribution of SPs in CPVI

There were 11 SPs during the de novo CPVI procedure, 10 (90.9%) of which were located at the left PV circular line (including 6 at the AS segment, 2 at the top, 1 at the PS segment and 1 at the bottom), and the remaining one at the AS aspect of the right PV circular line. Out of 11 SPs, 7 (63.6%) were located at the AS segment of the left PV circular line (Figure 2), which became the most common site of SP in high power CPVI procedure.

### 3.3. Comparison of Procedural Parameters

PV isolation was achieved in all SP and non-SP patients. The ablation procedural parameters at the SP sites and the non-SP matched sites are compared in Table 2. The RF power, average/maximal temperature, average/maximal CF and impedance at the end of ablation were similar between the two groups, while there was significantly shorter RF delivery duration, lower impedance at the beginning of ablation, greater Δimpedance before SP occurrence and lower AI in SP patients than those in non-SP patients.

### 3.4. Predictors for SPs Occurrence

**Figure 4:**
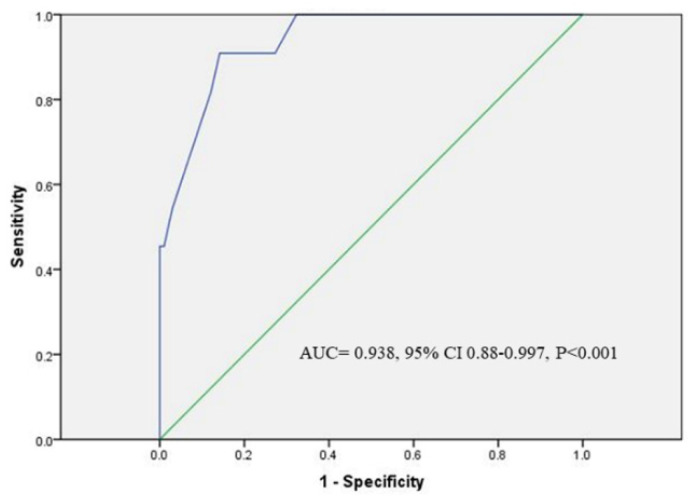
**Receiver operating characteristic (ROC) curve analysis for Δimpedance to predict SP occurrence.** Area under the curve (AUC) was 0.938, 95% confidence interval (CI) 0.88–0.997, *p* < 0.001.

Of all the procedural parameters in SP patients, Δimpedance was the only significant predictor for SP occurrence in high-power CPVI procedures by multivariate binary logistic regression analysis (OR 1.65, 95% CI 1.24–2.20, *p* = 0.001). ROC curve analysis determined the cut-off Δimpedance value of 12 Ω for prediction of SP (sensitivity 90.9%; specificity 85.9%; AUC = 0.938, 95% CI 0.88–0.997, *p* < 0.001, Figure 4). Immediately before SP occurrence, the impedance elevated in eight patients by 16.5 ± 5.9 Ω (4 (50%) impedance rise > 15 Ω), and decreased in three patients by 30 Ω, 20 Ω and 12 Ω, respectively.

### 3.5. Results of Catheter Tip Displacement by Two Means of Placement

For both means of catheter placement, 33 Tip_disp_ values were calculated in 11 non-SP control patients. The average Tip_disp_ was 3.2 ± 1.6 mm (range1.2–7.2 mm) for “PV-side of ridge” placement, which was significantly greater as compared to 2.0 ± 0.8 mm (range 0.5–3.7 mm) for “edge of ridge” placement, *p* < 0.001. For “edge of ridge” placement, there was no significant difference in Tip_disp_ values measured at 9:00, 10:30 and 12 o’clock direction of the LPV lesion line (1.7 ± 1.0 mm, 1.9 ± 0.7 mm, and 2.4 ± 0.7 mm, respectively, *p* = 0.14). For “PV-side of ridge” placement, there was comparable Tip_disp_ values at three sites of the LPV lesion line (3.4 ± 1.9 mm, 3.1 ± 1.8 mm and 3.1 ± 1.2 mm, respectively, *p* = 0.89) (more detail see in Supporting Information).

### 3.6. Complications

SPs were uneventful in all 11 patients, without the evidence of acute or delayed pericardial effusion by TTE detection. Groin hematoma in 1 non-SP patient was treated by mechanical compression. No silent or symptomatic cerebral embolism was detected during the peri-procedural period.

## 4. Discussion

Conventional non-irrigated RF ablation was associated with a higher risk of thrombus/char formation. Fifty-six-hole porous catheters facilitated to reduce the risks of thrombus and create lesions of bigger volume. However, due to the potent cooling effect of the surrounding saline irrigation, the temperature feedback from the tissue was inadequate, resulting in 59 SPs out of 226 low-power (27 W) CPVI procedures [9]. Incorporation of CF technology and AI algorithm significantly improved the efficacy and reduced SP occurrence in CPVI procedures [5,6].

Although 4 SPs out of 50 patients in the FAFA AI study were uneventful, tamponade occurred in 2 of 59 SPs in the previous study [7,9]. In view of this, the potential risk of SP should not be underestimated, and we applied less than 50 W for CPVI. In our study, SP occurred in only 11 (1.57%) out of 700 de novo high-power CPVI cases, and did not cause tamponade in any case, indicating the risk of SP might increase with the elevated RF power in CPVI.

In our study, RF duration was shorter and AI value was lower in SP patients than those in the control, indicating that AI value and RF duration were not reliable predictors for SP. A higher AI value could be obtained by lower power but longer-duration ablation; whereas a higher-power ablation resulted in early occurrence of SP before the higher AI value could be obtained [12].

Δimpedance immediately before SP had been under investigation in several studies [9,13,14]. Δimpedance > 15 Ω was found to be associated with increased risk of SP [13,14]. A recent ex vivo study reported that the percentage of delta impedance > 15% predicted SP occurrence [15]. In our study, Δimpedance > 12 Ω was found to be the only significant predictor for SP occurrence. These results propose the adoption of reasonable impedance cut-off settings to reduce the risk of SP.

In our study, SP occurred much more often at LPV ablation (10/11), and the AS segment of LPV circular line was the most common site of SPs. To the best of our knowledge, these results had not been reported in previous studies, and were of value for reminding us of the relatively high risk of SP at left-sided AS segment ablation.

Several factors were found to be associated with elevated occurrence of SP in two ex vivo studies [11,15]. Ablation in the pouch might predispose to the occurrence of SP [15]. Furthermore, tissue coverage, defined as the extent of electrode–tissue contact, could increase the incidence of SP from 0 for level I (16% coverage) to 100% for level III (100% coverage) under the same CF and force-time integral (FTI) [11]. These findings provided important clues for explaining the reasons of SP predilection at the left-sided AS segment.

It was well-recognized that the catheter tip was usually placed at the “PV-side” of the ridge in order to improve catheter stability at the AS segment of LPV, the only area where ablation was performed inside the PV’s ostium rather than at the PV’s antrum [10]. In our study we found the Tip_disp_ value for the “PV-side” placement was significantly greater than that for the “edge of ridge” placement, which indicated more prominent engagement with the PV wall and more tissue coverage over the catheter tip, the latter predisposed to SP occurrence at the AS segment (Figure 3). Hence it was advisable to place the catheter tip at the edge of the ridge for AS segment ablation, rather than at the conventional PV-side of the ridge, in order to reduce the risks of SP and PV stenosis as well. Of note, the Tip_disp_ was greater at the 10:30 o’clock direction as compared to that at the 9 o’clock direction even for “edge of ridge” placement, indicating that RF ablation at this area has a tendency toward SP occurrence even with desirable catheter placement.

## 5. Limitations

This study had several limitations. Firstly, although RF ablation at one site of the AS segment of LPV lesion line might cause SP occurrence, ablation at the neighboring sites of the same segment did not result in SP, even if the means of catheter placement and RF power delivery settings were unchanged. This phenomenon indicated the existence of some unknown mechanisms related to SP, and the “tissue coverage” assumption was merely one of the reasonable explanations. For instance, local unknown small pouch/frail tissue might pre-exist in rare cases and markedly facilitate SP occurrence. Tissue perfusion status might also have great impact on SP occurrence. However, the anatomic anomaly or tissue perfusion status could not be evaluated precisely ahead of ablation. In this scenario, SP occurrence could hardly be predicted in advance. Secondly, by comparison of the procedural parameters between the SP and non-SP patients, we found Δimpedance > 12 Ω could exclusively predict SP occurrence. However, the mechanisms underlying impedance change had not been clarified, especially for the lesions with short duration and low AI, and hence warrant further investigation. Thirdly, although the predictor for SP was found in this study, the predictors for cardiac perforation after SP occurrence were not explored, since all 11 SPs were fortuitously benign and eventless.

## 6. Conclusions

In conclusion, the prevalence of SP was 1.57% in high-power CPVI procedures with the most common site at the AS segment of the LPV circular line. Δimpedance > 12 Ω during RF energy delivery could exclusively predict SP occurrence. “PV-side” ablation at the AS segment might predispose to SP because of excessive tissue coverage.

## Figures and Tables

**Table 1:** The baseline demographic data in SP, non-SP and Tip_disp_ measurement group.

	SP Patients	Non-SP Patients	Tip_disp_ Measurement Patients
Number of cases	11	33	11
Age (years)	61.9 ± 12.6	63.0 ± 11.6	62.3 ± 10.8
Male, n (%)	7 (63.6)	21 (63.6)	7 (63.6)
Duration of AF (months)	4 (1, 24)	6 (1, 60)	11 (3, 12)
Comorbidities			
Hypertension, *n* (%)	7 (63.6)	17 (51.5)	7 (63.6)
Diabetes Mellitus, *n* (%)	0 (0)	6 (18.2)	4 (36.4)
Coronary artery disease, *n* (%)	1 (9.1)	6 (18.2)	0(0)
Heart failure, *n* (%)	5 (45.4) *	2 (6.1) *	1 (9.1)
History of stroke, *n* (%)	0 (0)	2 (6.1)	1 (9.1)
History of LAAC, *n* (%)	2 (18.2)	0 (0)	0 (0)
TTE measurement			
LAD (mm)	46.6 ± 4.9	46.2 ± 4.4	44.6 ± 6.2
LVEDD (mm)	51.6 ± 4.6	49.5 ± 5.8	47.1 ± 5.8
LVESD (mm)	36.6 ± 6.7	33.7 ± 6.1	30.1 ± 5.1
LVEF (%)	55.8 ± 13.0	60.2 ± 8.6	62.6 ± 8.7

* *p* < 0.05 compared between SP and non-SP group. TTE, transthoracic echocardiography; LAD, left atrial diameter; LVEDD, left ventricular end-diastolic diameter. LVESD, left ventricular end-systolic diameter; LVEF, left ventricular ejection fraction.

**Table 2:** Comparison of ablation parameters in SP and non-SP lesions at matched sites.

	SP Patients (*n* = 11)	Non-SP Patients (*n* = 33)	*p* Value
Number of lesions	11	99	
RF power (Watts)	40.4 ± 3.5	40.3 ± 2.8	0.87
RF energy delivery duration (s)	13.9 ± 6.3	23.3 ± 6.0	<0.001
Average temperature (°C)	22.2 ± 1.3	22.1 ± 2.0	0.98
Maximum temperature (°C)	24.9 ± 3.5	26.0 ± 3.0	0.25
Average contact force (g)	6.9 ± 1.8	6.4 ± 1.7	0.34
Maximum contact force (g)	12.8 ± 3.4	15.0 ± 5.3	0.19
Δimpedance (Ω)	17.6 ± 6.7	6.7 ± 4.1	<0.001
Impedance at the beginning of ablation (Ω)	112.7 ± 10.4	127.1 ± 15.7	0.004
Impedance at the end of ablation	119.1 ± 21.5	122.2 ± 14.0	0.5
AI value	357.7 ± 68.8	430.2 ± 30.7	<0.01

RF, radiofrequency; AI, ablation index.

## Data Availability

Authors can confirm that all relevant data are included in the article and/or its Supplementary Information Files.

## Supplementary Materials

The supporting information can be downloaded at: https://www.mdpi.com/article/10.3390/jcdd9120441/s1.
