# Supplementary material for: Prevalence, Predictors and Mechanisms of Steam Pops in Ablation Index-Guided High-Power Pulmonary Vein Isolation
Source: J Cardiovasc Dev Dis. 2022 Dec 7;9(12):441. doi: 10.3390/jcdd9120441 (PMC9781888; doi:10.3390/jcdd9120441)
Supplement: Supplementary file 1 [file jcdd-09-00441-s001.zip › jcdd-1989032-supplementary.pdf]

Supplemental Figure Legend

Typical techniques for determining the Tip_{loc} and calculation of the value of Tip_{disp}. **Panel A** and **B**, for the "edge of ridge" placement at the antero-superior segment of left PV, the Tip_{loc} was obtained at CF 5 g (blue dots) and 10 g (pink dots) gating at the end-expiratory phase, respectively; and was displayed as blue and pink dots on the geometry (LL and PA view). **Panel C** and **D**, for the "PV-side of ridge" placement at the same PV segment, the Tip_{loc} was acquired at CF 5 g and 10 g gating at the end-expiratory phase, respectively; and was displayed as blue and pink dots on the left atrial geometry (LL and PA view). **Panel E,** the six pairs of Tip_{loc} at 9, 10:30 and 12 o'clock direction of the left PV line were displayed on the modified PA view of the geometry. Tip_{disp} was defined as the distance between Tip_{loc} at CF 10 g and Tip_{loc} at CF 5 g. In this patient, it was 4.1 mm for the "PV-side of ridge" placement and was 3.0 mm for the "edge of ridge" placement at 9 o'clock direction of the left PV line. *Tip_{loc}, Tip location; Tip_{disp}, Tip displacement; CF, contact force; PV, pulmonary vein; LL, left lateral; PA, postero-anterior; ABL, the ablation catheter; LSPV, left superior PV; LIPV, left inferior PV; RSPV, right superior PV; RIPV, right inferior PV; LAA, left atrial appendage.*

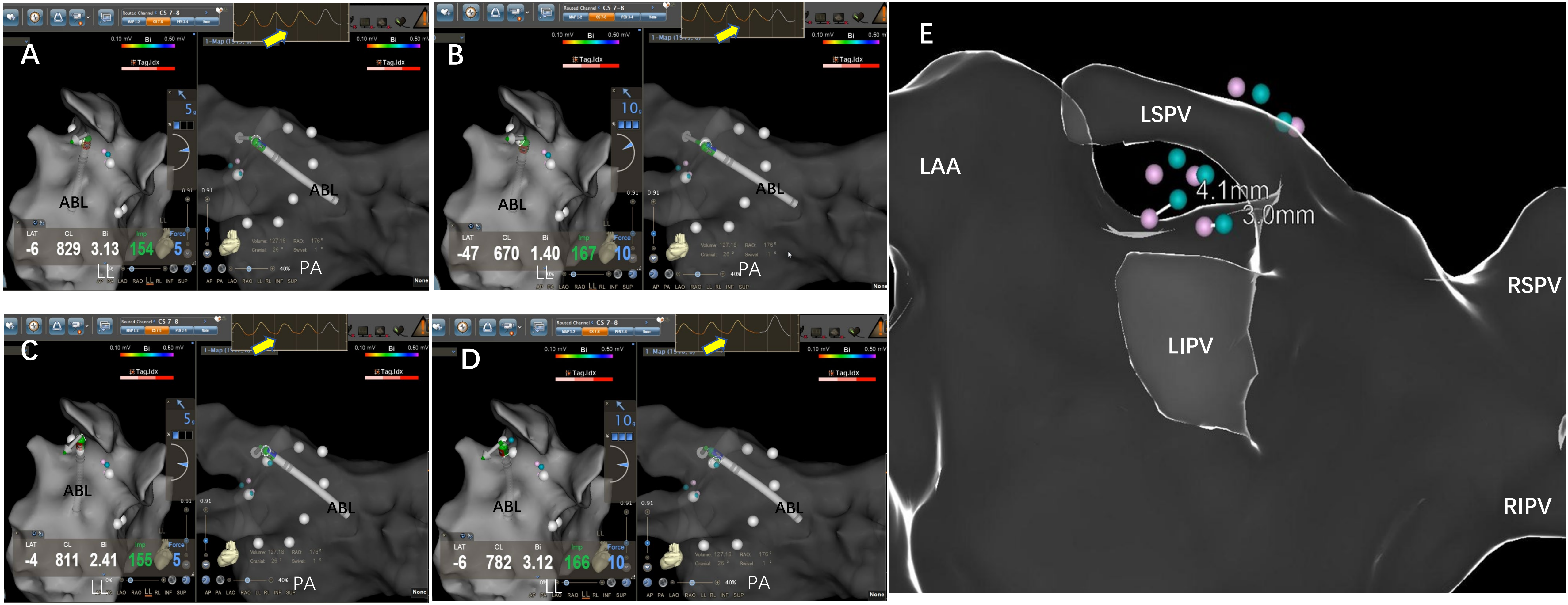

Supplemental Figure S1
